# The Influence of Graphene Oxide Composition on Properties of Surface-Modified Metal Electrodes

**DOI:** 10.3390/ma15217684

**Published:** 2022-11-01

**Authors:** Natalia Festinger, Aneta Kisielewska, Barbara Burnat, Katarzyna Ranoszek-Soliwoda, Jarosław Grobelny, Kamila Koszelska, Dariusz Guziejewski, Sylwia Smarzewska

**Affiliations:** 1 Łukasiewicz Research Network-Lodz Institute of Technology, Maria Skłodowska-Curie 19/27, 90-570 Lodz, Poland; 2 Department of Materials Technology and Chemistry, Faculty of Chemistry, University of Lodz, Pomorska 163, 90-236 Lodz, Poland; 3 Department of Inorganic and Analytical Chemistry, Faculty of Chemistry, University of Lodz, Tamka 12, 91-403 Lodz, Poland

**Keywords:** gold electrode, platinum electrode, graphene oxide, reduced graphene oxide, voltammetry

## Abstract

The present paper describes the effect of the concentration of two graphene oxides (with different oxygen content) in the modifier layer on the electrochemical and structural properties of noble metal disk electrodes used as working electrodes in voltammetry. The chemistry of graphene oxides was tested using EDS, FTIR, UV–Vis spectroscopy, and combustion analysis. The structural properties of the obtained modifier layers were examined by means of scanning electron and atomic force microscopy. Cyclic voltammetry was employed for comparative electrochemical studies.

## 1. Introduction

Electrochemical methods are based on measurement of the current associated with the molecular properties and interfacial processes of chemical species. The recorded response results from direct transformation of the desired chemical information (concentration, activity) into a current signal (potential, current, resistance, or capacity), according to the selected method. Voltammetry is considered as one of the most sensitive electroanalytical methods, suitable for the determination of trace amounts of many metals and compounds in clinical, industrial, and environmental samples [1,2,3,4]. Various voltammetric techniques provide a wealth of chemical, electrochemical, and physical information, such as quantitative analysis, diffusion and reaction rate constants, and number of electrons involved in redox reactions [5,6]. The effectiveness of voltammetric procedures is strongly influenced by the working electrode material [7,8]. The working electrode should provide a high signal-to-noise ratio as well as reproducible signals. Thus, electrode selection depends mainly on the redox behavior of the target analyte, electrical conductivity, surface reproducibility, and background current over the potential window required for measurement. A range of materials have found application as working electrodes in electroanalysis. The most popular types contain mercury, carbon, or noble metals [8]. Among the noble metals, platinum and gold are the most widely used for metallic electrodes, as they offer very favorable electron transfer kinetics and a large available potential range [8]. In contrast, the low hydrogen overvoltage on those electrodes limits the cathodic potential window. Additionally, high background currents associated with the formation of surface oxides or adsorbed hydrogen layers can cause problems. Such films can also strongly alter the kinetics of electrode reaction, leading to irreproducible data [8]. Compared with platinum electrode, the gold one is more inert, and hence, less prone to the formation of stable oxide films or surface contamination. The abovementioned difficulties can be addressed by modifying the surface of platinum and gold electrodes with a specific modifier layer. Still, noble metals used as starting materials for electrodes are well-known for their versatile usage and applications, also in sensing and as catalysts [9,10]. The occurrence of stable oxide films at the electrode surface together with enhanced physical and electrocatalytical properties tuned by the presence of graphene oxides makes this combination a primary choice for electroanalytical purposes.

Graphene, which consists of a one-atom-thick planar sheet containing an sp2-bonded carbon structure with exceptionally high crystalline and electronic quality, is a novel material that has emerged as a rapidly rising star in the field of material science [11,12]. Ever since its discovery in 2004 [13], graphene has been making a profound impact in many areas of science and technology due to its remarkable physicochemical properties. These include a high specific surface area [14], extraordinary electronic properties and electron transport capabilities [15], unprecedented pliability [16] and impermeability, high mechanical strength [17], and excellent thermal and electrical conductivity [18]. One branch of graphene research deals with graphene oxide (GO), which is a precursor in graphene synthesis by either chemical or thermal reduction processes. One of the advantages of graphene oxide is easy dispersibility in water and other organic solvents, as well as in different matrices, due to the presence of oxygen functionalities. This property is very important when it is mixed with other materials with a view to improving their electrical and mechanical properties. On the other hand, in terms of electrical conductivity, graphene oxide is often described as an electrical insulator due to the disruption of its sp2 bonding networks. In order to recover the honeycomb hexagonal lattice—and with it, electrical conductivity—graphene oxide must be reduced. However, once most oxygen groups are removed, the obtained reduced product is more difficult to disperse due to its tendency to aggregate. It is worth noting that graphene oxide and graphene have attracted exceptional attention from the scientific community. Concerning 2022 (data for 15 July 2022), a keyword query in the Scopus database revealed 12,194 and 4258 research papers concerning graphene and graphene oxide, respectively. Those publications can certainly be very inspiring, but also frustrating, if other research teams fail to reproduce the results. A lack of research reproducibility has always been a major issue in the scientific community. With graphene oxide and reduced graphene oxide, the situation is very complicated. While each single carbon layer containing oxygen groups is called graphene oxide, material obtained after GO reduction is called reduced graphene oxide (smaller quantity of oxygen groups after GO reduction is usually confirmed by spectroscopic measurements). Indeed, it cannot be excluded that an oxide synthesized in one laboratory as graphene oxide is structurally similar to reduced graphene oxide obtained by another research team. A similar problem occurs when purchasing graphene oxide from different suppliers. This is attributable to the fact that the precise atomic structure of GO still remains uncertain and perfect stoichiometry has never been achieved [19]. The study of GO structure is derived from the structural analysis of graphite oxide. Over the years, considerable efforts have been directed toward understanding the structure of that compound with the result that several conflicting explanations have been successively proposed. In 1939, Hofmann and Holst [20] developed a simple model in which graphite oxide was thought to consist of planar carbon layers modified with an epoxy (1,2-ether) group, with an overall molecular formula of C_2_O. Seven years later, Ruess [21] suggested that the carbon layers were not in fact planar but puckered and that the oxygen-containing groups were hydroxyl and ether-like oxygen bridges, randomly distributed on the carbon skeleton. In order to account for the acidic properties of graphite oxide, Hofmann [22] proposed enol- and keto-type structures, which also contained hydroxyls and ether bridges. In 1969, Scholz and Boehm [23] presented a GO model in which epoxide and ether groups were completely replaced by carbonyl and hydroxyl groups. According to Nakajima et al. [24], GO consisted of two carbon layers linked to each other by sp3 carbon−carbon bonds. Szabó [25] proposed a new structural model that involves a carbon network consisting of trans-linked cyclohexane chairs and ribbons of flat hexagons with C=C double bonds as well as functional groups such as tertiary OH, 1,3-ether, ketone, quinone, and phenol (aromatic diol). One of the few common features of all the published models is the presence of various oxygen-containing functional groups in GO. It is known that oxygenated groups (e.g., hydroxyl, epoxy, carboxyl, carbonyl, phenol, lactone, and quinone) can strongly affect the electronic, mechanical, and electrochemical properties of GO [26,27,28]. In recent years, research on GO-based materials has been extensive, particularly with respect to their electrochemical applications [29,30,31,32,33,34]. In such studies, GO was produced by different methods, which were sometimes applied interchangeably as it was assumed that they all led to the same reaction product. Nowadays, it is known that the type and quantity of oxygen groups depends largely on the synthesis method. GO is generally produced by synthesis with concentrated H_2_SO_4_ along with: (1) sodium nitrate for in situ production of nitric acid in the presence of KMnO_4_ (Hummers’ method); (2) fuming nitric acid and a KClO_3_ oxidant (Staudenmaier method); (3) concentrated phosphoric acid with KMnO_4_ (Tour method); or (4) concentrated nitric acid and a KClO_3_ oxidant (Hoffmann method) [35,36,37,38]. However, the obtained oxides differ significantly in the number of oxygen-containing groups (C/O ratio) as well as in terms of the types of oxygenated carbon bonds present. Generally speaking, oxides prepared using KClO_3_ as an oxidant (Staudenmaier’s and Hofmann’s methods) exhibit a higher C/O ratio, whereas methods employing KMnO_4_ (Hummers and Tour) yield a larger proportion of oxygen-containing groups. Oxidative methods employing KClO_3_ result in oxides containing mostly CO groups (hydroxyl, epoxy), whereas methods using KMnO_4_ lead to oxides containing large amounts of carbonyl (Hummers) or both carbonyl and carboxyl groups (Tours) [39]. 

The aim of this study was to compare the properties of noble metal electrodes modified with graphene oxide and to determine how graphene oxide chemical composition and concentration affect their electrochemical and structural (morphology, topography, roughness) properties.

## 2. Materials and Methods

### 2.1. Apparatus and Solutions

The surface topography of the studied electrodes was investigated using an atomic force microscope (AFM, Dimension Icon, Bruker Corporation, Billerica, MA, USA). The AFM measurements with the scan size of 5 µm × 5 µm were performed in the tapping mode using silicon scanning probe (TESPA-V2, Bruker AFM Probes) with a nominal spring constant of 42 N/m and resonance frequency of 320 kHz. The roughness parameters Ra and Rq were defined on the basis of AFM topography images (average values taken from 256 surface profiles). The surface morphology and elemental composition of electrodes was investigated with high-resolution scanning electron microscopy (Nova NanoSEM 450, FEI, Hillsboro, OR, USA) equipped in a Schottky field emission electron emitter. The surface morphology measurements were performed using CBS detector, enabling the observation of surface using signal of backscattered electrons (BSE). The images were recorded at 3 kV with electron beam deceleration 4 kV, spot size 2.5, and working distance 6.7 mm. The chemical elemental analysis of GO I- and GO II-modified gold electrodes was determined with EDAX Roentgen spectrometer (EDS) with Octane Pro Silicon Drift Detector (SDD) (AMELTEK, Berwyn, PA, USA). Attenuated total reflection Fourier transform infrared (ATR–FTIR) spectra were recorded on a Nicolet iS50 spectrometer equipped (Thermo Scientific, Madison, WI, USA)with an MCT detector and the GATR accessory with Ge crystal over the spectral region from 600 to 4000 cm^−1^ with a resolution of 4 cm^−1^. Electroanalytical measurements were carried out using a µAutolab instrument (EcoChemie, The Netherlands) controlled by GPES 4.9 electrochemical software. The three-electrode electrochemical cell employed in the study consisted of a reference electrode, an auxiliary electrode (platinum wire), and a working disk electrode (platinum or gold—please notice that experiments with platinum and gold electrodes are performed with different potential windows).

The potential of the working electrode was measured vs. an Ag/AgCl electrode. Double-distilled water was used throughout the experiments. All the chemicals, including graphene oxide (GO I), reduced graphene oxide (GO II), and hexacyanoferrate system were purchased from Sigma-Aldrich and used as received.

The GO I/GO II suspension was prepared weekly by dispersing an appropriate amount of GO I/GO II powder in DMF (dimethylformamide) in a 5 mL volumetric flask. The resultant solution was kept in a refrigerator at 4 °C in the dark. Spectrophotometric measurements were made using a Cary 100 Bio UV–Vis spectrophotometer (Agilent, Santa Clara, CA, USA).

### 2.2. Measurement Procedure

The general procedure used to obtain voltammograms was as follows: Ten mL of supporting electrolyte was placed in the voltammetric cell and the solution was purged with argon for 10 min (if necessary). After recording the initial blank, the required volumes of the analyte were added by means of a micropipette. Then, the solution was deoxygenated for 10 s (if necessary), and a voltammogram was recorded. All electrochemical measurements were carried out at ambient temperature. Each measurement was repeated three times and a mean was calculated.

In order to obtain FTIR spectra of both graphene derivatives, DMF solutions of GO I and GO II at a concentration of 20.0 g L^−1^ were prepared. Afterwards, 20 µL of each solution was deposited on alumina foil by sessile drop technique and, subsequently, the samples were allowed to evaporate the solvent. Final spectra were received by the addition of 64 scans at a resolution of 4 cm^−1^.

### 2.3. Preparation of Working Electrodes

Modifying solutions containing graphene oxides were prepared with dimethylformamide. Suspensions at concentrations ranging from 1.0 to 50.0 g L^−1^ were prepared for each oxide. Prior to modification with GO I or GO II, the working electrode surface was sonicated in ethanol for 60 s. Next, working electrode surface was mechanically polished with 0.05 μm Al_2_O_3_ slurry on a polishing cloth to a mirror finish. Then, it was ultrasonically treated in ethanol for 180 s and washed with double-distilled water. The modifier suspension (3 µL) was dropped onto the surface of the cleaned electrode and dried at ambient temperature. A new modifier film was prepared before each series of measurements.

## 3. Results and Discussion

### 3.1. Electrochemical Studies

**Figure 1:**
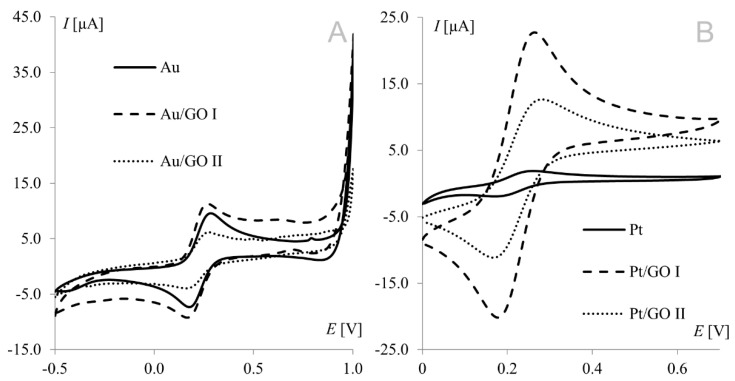
Voltammograms of 1 mM Fe(CN)_6_^3−/^Fe(CN)_6_^4−^ recorded from bare, graphene oxide I-, and graphene oxide II-modified working electrodes ((**A**) gold electrode, (**B**) platinum electrode); scan rate, 50 mV s^−1^; supporting electrolyte, 1 M KCl.

Voltammetric measurements provide a wealth of information concerning the properties and characteristics of electrochemical processes. Cyclic voltammetry is an important and widely used technique determining analyte electrochemical behavior, including the formal redox potential, thermodynamic and transport properties, electron transfer kinetics, and adsorption processes. The hexacyanoferrate(II)/(III) redox couple undergoes a nearly reversible electrode reaction without any complications of preceding or post-chemical reactions; so, it has been a popular choice as a redox standard in CV. Electrochemical measurements were performed at modified working electrodes using the Fe(CN)_6_^3−^/Fe(CN)_6_^4−^ redox couple and suspensions of graphene oxides (GO I and GO II) in a concentration range from 1.0 to 50.0 g L^−1^. Figure 1 shows a voltammogram of the Fe(CN)_6_^3−^/Fe(CN)_6_^4−^ redox couple recorded on bare and modified gold (A) and platinum (B) electrodes. Both working electrodes were modified with graphene oxide suspensions at a concentration of 1.0 g L^−1^.

**Figure 2:**
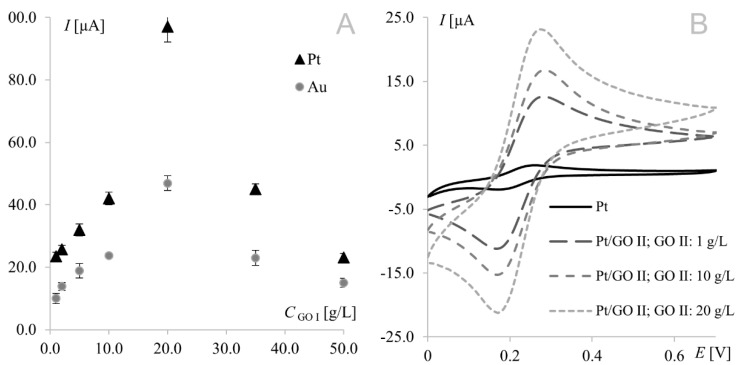
(**A**) Relationship between 1 mM Fe(CN)_6_^4−^ anodic peak current recorded at the platinum/gold electrode modified with graphene oxide I suspensions prepared in dimethylformamide and GO I concentration (GO I concentrations from 1 to 50.0 g L^−1^). (**B**) Voltammograms of 1.0 mM Fe(CN)_6_^3−/^Fe(CN)_6_^4−^ recorded at the bare platinum electrode and at the electrode modified with graphene oxide II; scan rate, 50 mV s^−1^; supporting electrolyte, 1 M KCl.

The strongest signals at both electrodes were observed for the electrode surface-modified with GO I. Significant difference may be observed for GO II suspension, which influence tested working electrodes in the opposite way. As can be seen in Figure 1, model redox system signals recorded on platinum electrode modified with GO II exhibit very good morphology and increased current in comparison with bare electrode. At gold electrodes, such modification led to signals that were weaker even than those recorded at the bare electrode. This suggests that some concentrations of the modifier layer may block the electrode surface causing significant deterioration of analytical parameters. According to this, additional experiments were made for Au electrode and it was concluded that such behavior is observed for GO II suspension concentration 1.5 g L^−1^ and lower. The relationships between hexacyanoferrate anodic peak current and the surface concentration of GO I recorded at both Au and Pt electrodes are shown in Figure 2A. As can be seen, both dependencies have a similar course, with a maximum for electrodes modified with 20.0 g L^−1^ GO I suspension. An analogous study using GO II suspensions also revealed the strongest signals of the Fe(CN)_6_^3−^/Fe(CN)_6_^4−^ redox couple for the 20.0 g L^−1^ concentration at both Pt and Au electrodes. Figure 2B shows Fe(CN)_6_^3−^/Fe(CN)_6_^4−^ voltammograms recorded at the GO II-modified platinum electrode.

**Figure 3:**
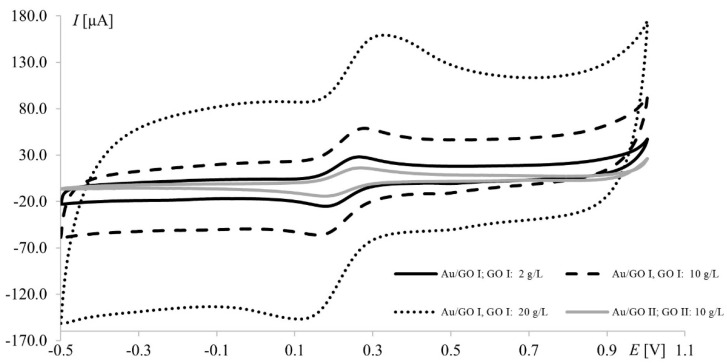
Voltammograms of 1.0 mM Fe(CN)_6_^3−^/Fe(CN)_6_^4−^ recorded at the gold electrode modified with graphene oxides I and II; scan rate, 75 mV s^−1^; supporting electrolyte, 1 M KCl.

It is worth noting that the highest currents recorded at electrodes modified with 20.0 g L^−1^ GO II (17 μA and 14 μA at Pt and Au, respectively) were much lower than those obtained at electrodes modified with 20.0 g L^−1^ GO I (97 μA and 47 μA for Pt and Au, respectively). Since GO II was purchased as reduced graphene oxide and RGO has fewer oxygen groups (than GO), which improves its electrical conductivity, the opposite results were expected (oxygen content in both GOs will be thoroughly discussed in Section 3.3). However, this should be discussed not only from the point of view of peak current but also the shape of the voltammograms (Figure 3). In voltammetry, the current signal is produced by two different currents: the first one (faradic) corresponds to analyte oxidation or reduction, with its magnitude depending on analyte concentration in solution and all kinetic steps occurring at the electrode (electron-transfer process); the second one (capacitive) is generated by the “electric double layer” at the electrode–solution interface and, as such, is unrelated to the electron transfer process. As it is impossible to completely separate the two types of current, techniques and electrodes minimizing the influence of capacitive current are sought. As can be seen in Figure 3, gold electrodes modified with GO I do not seem to meet these requirements. The characteristic rectangular shape of voltammograms recorded on GO I layer is a favorable and desirable phenomenon in high-performance supercapacitors research but not in electroanalysis [40]. It is worth noting that such capacitive performance is connected with partial restoration of π-conjugation structure and improved electronic conductivity. Both mentioned features are characteristic for reduced grapheme oxide. 

**Figure 4:**
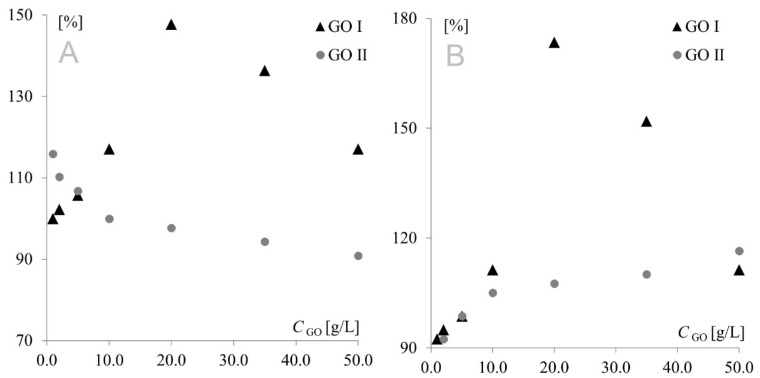
Signal separation measured for Au (**A**) and Pt (**B**) electrodes modified with GO I and GO II.

The model redox pair Fe(CN)_6_^3−/^Fe(CN)_6_^4−^ is a chemically reversible system, in which the oxidized form of the solution species can be regenerated from the reduced form (and vice versa), and both forms are stable on the time scale of the voltammetric experiment. However, after preliminary studies, a negative impact of modifications on electrochemical reversibility could not be excluded. If this was the case, the electron transfer would be so slow that the peak potential would not reflect the equilibrium activity of the redox couple at the electrode surface. To determine whether GO modification had a positive or negative effect, cathodic and anodic signal separation (difference between cathodic and anodic peak potentials) was analyzed. In the theoretical model, the difference between the anodic and cathodic peak potentials (*E_a_* and *E_c_*, respectively) should be equal to 59 mV/n for fully electrochemically reversible systems. However, the majority of redox systems used are quasi-reversible, with anodic/cathodic separation being much higher than 59 mV, even for fast quasi-reversible redox pairs. The separations measured in the present study are shown in Figure 4. To make the figure clearer, the separation obtained for the bare electrode was used as reference (100%).

**Figure 5:**
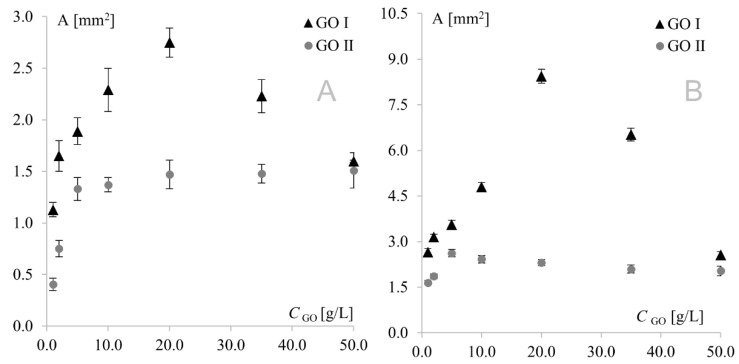
Electroactive surface of Au (**A**) and Pt (**B**) electrodes modified with GO I and GO II.

As can be seen, similar behavior was observed for Au and Pt electrodes modified with GO I. In this case, modifier concentration had a very strong influence on peak current; therefore, it was also the predominant factor affecting separation. This fact precluded investigation of the impact of the remaining factors on separation, and consequently, on the electrochemical reversibility of the system. A different behavior was observed for GO II, where signal intensity (with the highest currents also observed for the 20 g L^−1^ concentration) was only one of several factors influencing peak separation. At the platinum electrode, the lowest separation values were observed for the lowest modifier concentrations, while at the gold electrode, the highest concentrations afforded the best reversibility. This shows again that GO I and GO II act like two totally different compounds. Another important diagnostic characterizing an electrode reaction is peak potential (*E_p_*). At fast electron transfer rates, *E*_p_ is independent of the scan rate, indicating a reversible electrode reaction. The influence of the scan rate was tested in the range of 10–500 mV s^−1^. It was found that the scan rate did not have any effect on peak potential (except for changes resulting from signal increment), indicating a quasi-reversible electrode reaction. The next very important diagnostic tool consists of the relationships log *I*_p_ vs. log *v* and *I*_p_ vs. *v*^1/2^. For both graphene oxides, linear plots of log *I*_p_ vs. log *v* were obtained with slopes of approx. 0.5, indicating an electrode reaction with the rate governed by diffusion of the electroactive species to an electrode surface. A linear relationship (for both electrodes with both graphene oxides) was also observed for *I*_p_ vs. *υ*^1/2^, which confirmed that the mass transport rate of the electroactive species to the electrode surface occurred across the concentration gradient. In such a case, the peak current *I*_p_ is governed by the Randles–Sevcik equation: *I*_p_ = *k n*^3/2^ A D^1/2^ C* *υ*^1/2^, where the constant *k* = 2.72 × 10^5^; *n* is the number of moles of electrons transferred per mole of the electroactive species; A is electrode area; D is the diffusion coefficient; C* is solution concentration; and *v* is the scan rate. The above formula was used to calculate the electroactive area of the Au and Pt electrodes modified with both graphene oxides (Figure 5). From an electrochemical point of view, observed dependence for GO I may be easily combined with observed peak currents—the bigger the electroactive surface, the higher the peak currents. The GO II case is much more complicated. For GO II, the highest current of model redox system was also observed for 20.0 g L^−1^ but the biggest electroactive surface was observed for lower concentrations. This suggest that observed currents are strongly connected with GO II chemical composition and the various oxygen functional groups present in its structure. Detailed studies on structural differences between GO I and GO II are described in Section 3.3.

### 3.2. Microscopic Analysis

**Figure 6:**
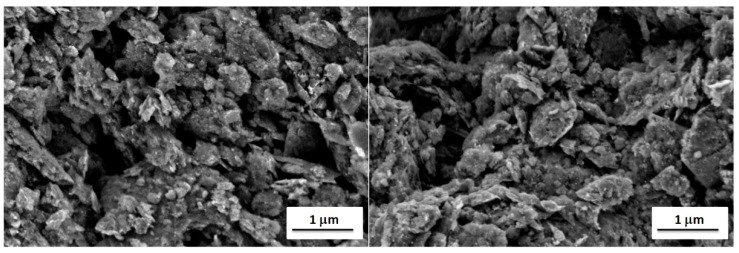
SEM images of gold electrodes with GO I (**left**) and GO II (**right**).

It is well known that the properties of a broad range of materials and the performance of a large variety of devices depend strongly on their surface characteristics [41]. Therefore, surface analyses were performed using scanning electron microscopy and atomic force microscopy to explain observed differences in electrochemical properties of fabricated graphene-oxide-modified electrodes. The first method allows to investigate the surface morphology of the modified electrodes, while the latter one allows to study their surface topography and roughness. Thus, their combination is very often used for surface characterization of the carbon electrodes [42,43]. Scanning electron microscope (SEM) images of GO I- and GO II-modified gold electrodes (suspension concentration 20.0 g L^−1^) are shown in Figure 6. The comparison of the SEM images do not revealed significant differences in the morphology of samples. In both cases, a lot of irregularly distributed aggregates containing entangled graphene flakes can be observed. The distribution of aggregates on the electrode surfaces is random and homogenous. The size of aggregates is in the range of tens and hundreds of nanometers up to micrometers. The graphene aggregates form a continuous mesoporous layer on the surface of both electrodes, having a pore size in the range of hundreds nm. Moreover, the presence of graphene aggregates results in the high surface roughness and large surface area of both GO I- and GO II-modified gold electrodes.

**Figure 7:**
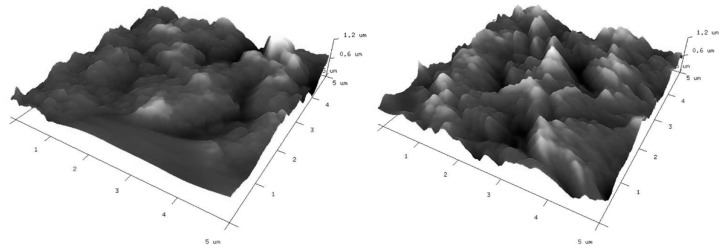
Three-dimensional AFM images of gold electrodes modified with graphene oxides: GO I (**left**) and GO II (**right**) suspensions.

As mentioned above, the modified electrodes were also characterized by atomic force microscopy (AFM) to give insight into their surface topography. Three-dimensional (3D) views of the graphene-oxide-modified gold electrodes are given in Figure 7. Corresponding two-dimensional (2D) AFM images with cross section profiles are presented in Figure S1. The surface characteristics of the investigated electrodes observed from AFM images are consistent with their SEM images. The applied modifications do not significantly differ in topographies. In both cases, a lot of irregularly distributed aggregates can be observed. There are also free spaces clearly visible between them. Roughness parameters (Rq and Ra) have been calculated from AFM images and are summarized in Table 1. From the presented results, it is clear that the roughness of the GO II-modified electrode is a little bit higher than that for the GO I-modified electrode. 

### 3.3. GO I and GO II Chemical Composition Analysis

**Figure 8:**
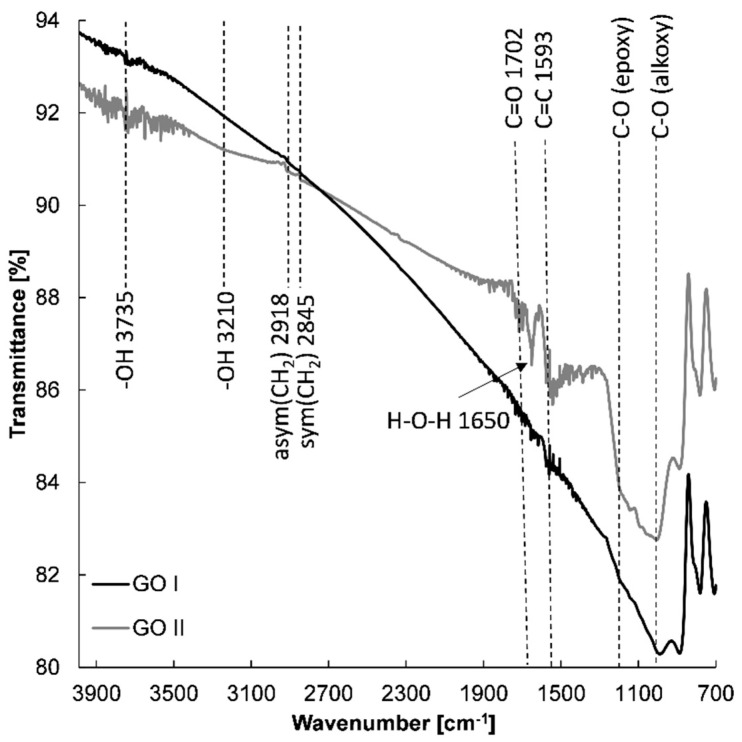
FTIR spectra of GO I and GO II.

With the aim of determining whether the chemical structure affects the electrochemical properties of the prepared graphene-based electrodes, spectroscopic measurements were conducted. Figure 8 shows the FTIR spectra of GO I and GO II nanostructures. FTIR spectroscopy provided evidence of the presence of various types of oxygen functional groups such as O-H, C=O, C-O, -O-, and C-OH on the GO, which could be located on the basal planes and edges of the GO flakes [44,45,46]. Small aggregated sharp peaks around 3735 cm^−1^ and a broad peak of very low intensity at 3210 cm^−1^ can be assigned to the stretching mode of the O–H bond, which reveal the presence of hydroxyl groups in graphene oxide [47,48]. Moreover, the peak at 1650 cm^−1^ can be designated to the stretching and bending vibration of water molecules adsorbed on graphene oxide [47]. The FTIR spectra of GO I and II also demonstrate the presence of other oxygenated functional groups with absorption peaks at 1050 cm^−1^ (alkoxy C-O) [44,46,49,50] and 1200 cm^−1^ (epoxide C-O-C or phenolic C-O-H stretch) [44,46,49,50]. It was found that in the case of GO I, absorption bands of all oxygen functional groups were less intense compared with GO II. Further insight into the graphene structure of GO I and GO II revealed other differences. Firstly, more epoxy groups are present on GO II in comparison with GO I. Secondly, the appearance of an absorption band at 1702 cm^−1^ confirms the presence of the C=O from carboxyl and/or carbonyl groups in the GO II structure [44]. The FTIR spectra indicate that GO I is more reduced than GO II. 

This was also confirmed by UV–Vis spectroscopy (Figure S2). UV–Vis spectra of GO I and GO II aqueous suspensions show only one absorption band at 270 nm. This peak is characteristic of reduced graphene oxide and corresponds to the π → π* transition of the C-C bond of the hexagonal carbon ring [51]. Moreover, the presence of this peak indicates the removal of oxygen-containing functional groups by reduction process and the restored electronic conjugation within graphene flakes [51]. However, the difference in the degree of GO I and GO II reduction can be seen in the UV–Vis spectra. Due to the increase in light absorption over the whole spectral region for GO I, it can be assumed that GO I is more reduced than GO II [46]. Another evidence of the higher reduction level of GO I is the lack of an absorption band at around 300 nm, which is attributed to n → π* transition of C=O bond of edged carboxyl group on graphene oxide [51]. Finally, spectroscopic measurements results were confirmed by combustion analysis (Table 2), which also showed that GO I is more reduced than GO II.

In the last step, modified electrodes were examined with Energy Dispersive X-ray Spectrometry. The EDS measurements revealed changes in the carbon-to-oxygen ratio in GO I and GO II samples (Table 3). The quantitative EDS element mappings of GO I and GO II are shown in Supplementary Material (Figures S3 and S4). Quantitative analysis shows that GO I had an oxygen content of 11.32 at.% and the atomic ratio of carbon to oxygen was 7.8, while in GO II the oxygen content was 16.62 at.% and the C/O ratio was about 5. These results indicate more oxygen content in the case of GO II compared with GO I and again suggests that GO I is more reduced than GO II. 

## 4. Conclusions

In this paper, the electrochemical and structural characteristics of two graphene oxides used as electrodes modifiers were analyzed. AFM and SEM results showed that the topographies of the investigated electrodes/modifications do not significantly differ. However, the EDS elemental analysis revealed changes in the oxygen content. This was also confirmed by combustion analysis, FTIR, and UV–Vis spectroscopy. Unexpectedly, more oxygen content was found in the case of GO II (declared as RGO) compared with GO I (declared as GO). From the electrochemical point of view, the presence of RGO (here, GO II) should improve the electrochemical conductivity; however, the opposite results were obtained. The detailed electrochemical studies with model redox system showed that purchased graphene oxides act as utterly different modifiers. Although basic analytical characteristics are similar—for example, the diffusional nature of registered signals remained unchanged—other more important properties are completely different (peak currents, peak separation, electroactive area, etc.). As a result, we may obtain two completely different sensors that could, but should not, be called uniformly “graphene-oxide-modified”.

Based on the obtained results, we can generally conclude that GO I is more reduced than GO II, which is the exact opposite of the general conception of graphene oxide structure. Hence, the following question arises: is there a chance to achieve good reproducibility among the scientific community using the not well-established uniform structure of GO? Undoubtedly, we may find some positive aspects of this graphene oxide case because by using graphene oxides of slightly changed chemical composition we can develop sensors with tailored properties towards a given analyte. On the other hand, we have to be aware of the negative aspect, i.e., the lack of reproducibility between “graphene-oxide-modified sensors” when being developed from diverse research teams. 

In conclusion, the authors believe that there is an urgent need to standardize such popular nanomaterials by assigning a CAS Registry Number to them. The orderliness in graphene oxides nomenclature will greatly shorten the search for appropriate graphene oxide and will contribute to improvements in electrochemical analysis. 

## Figures and Tables

**Table 1:** Surface roughness parameters calculated from AFM images.

	GO I	GO II
Rq	142.5 ± 5.3	184.0 ± 19.1
Ra	104.0 ± 2.4	144.3 ± 14.4

**Table 2:** Combustion analysis results (mass percent).

Element	GO I	GO II
C	85.44 ± 0.24	81.43 ± 0.13
H	0.380 ± 0.020	0.390 ± 0.059

**Table 3:** The content of carbon and oxygen in GO I and GO II determined by EDX measurements.

	GO I	GO II
C atomic%	88.68	83.38
C weight%	85.47	79.02
O atomic%	11.32	16.62
O weight%	14.53	20.98

## Data Availability

The data presented in this study are available upon request from the corresponding author.

## Supplementary Materials

The following supporting information can be downloaded at https://www.mdpi.com/article/10.3390/ma15217684/s1. Figure S1. 2D AFM images and profiles of gold electrodes modified with graphen oxides: GO I (left) and GO II (right) suspensions in concentration of 20 g L^−1^; Figure S2. UV-Vis spectra of GO I and GO II aqueous suspensions; Figure S3. The quantitative EDS element mapping of GO I (a); carbon C K (b); oxygen O K (c) and EDS spectrum (d); Figure S4. The quantitative EDS element mapping of GO II (a); carbon C K (b); oxygen O K (c) and EDS spectrum (d).
