# Supplementary material for: The Influence of Graphene Oxide Composition on Properties of Surface-Modified Metal Electrodes
Source: Materials (Basel). 2022 Nov 1;15(21):7684. doi: 10.3390/ma15217684 (PMC9654030; doi:10.3390/ma15217684)
Supplement: Supplementary file 1 [file materials-15-07684-s001.zip › materials-1854320-supplementary.pdf]

Supplementary data

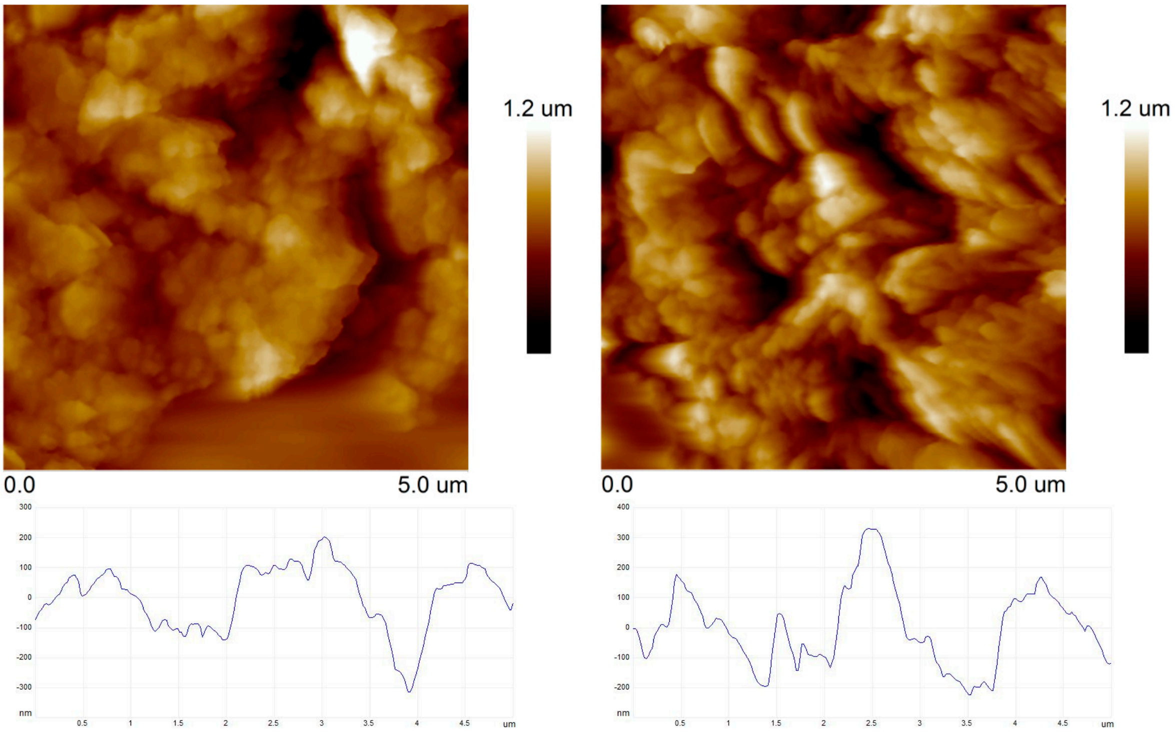

Fig. S1 2D AFM images and profiles of gold electrodes modified with graphen oxides: GO I (left) and GO II (right) suspensions in concentration of 20 g L^{-1}

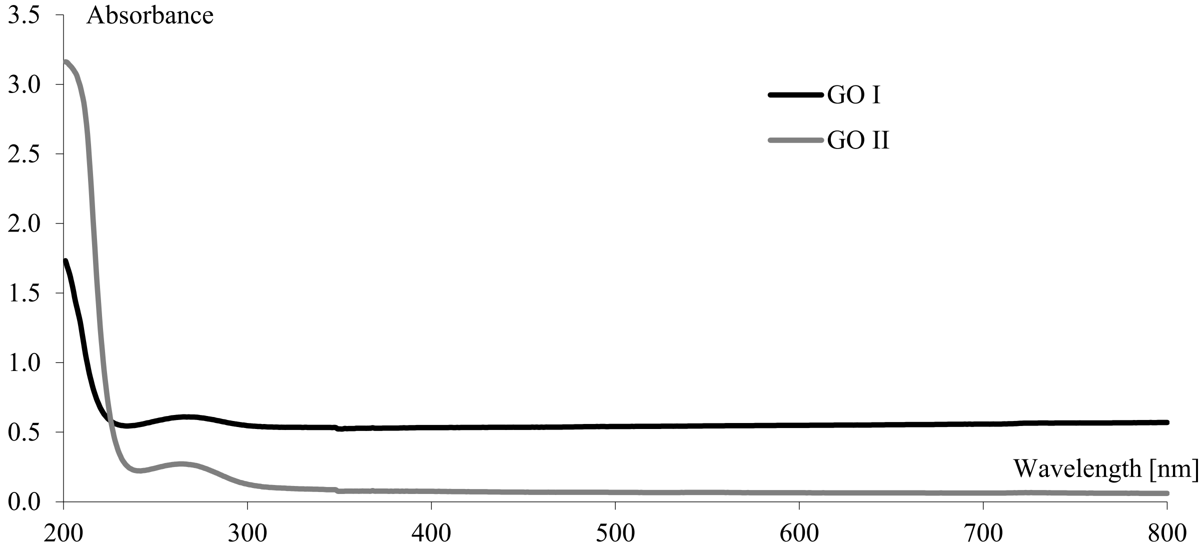

Fig. S2 UV-Vis spectra of GO I and GO II aqueous suspensions

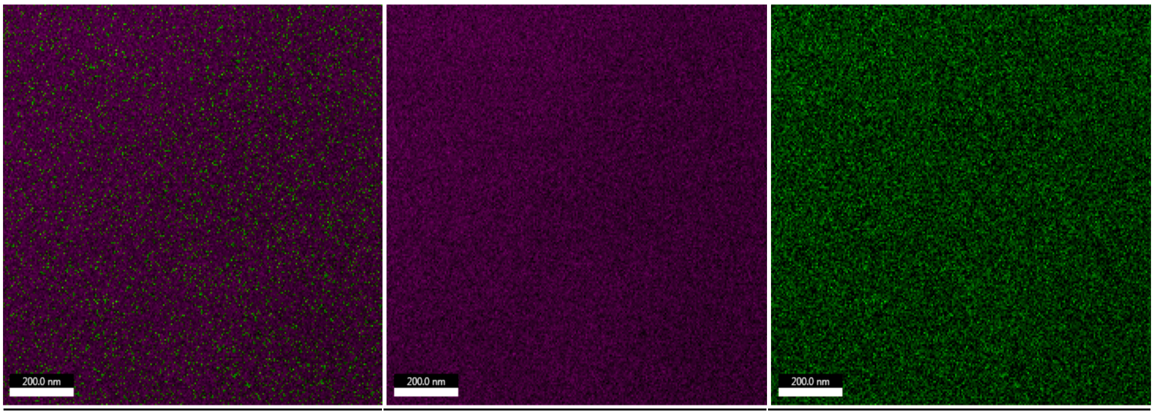

(a) (b) (c)

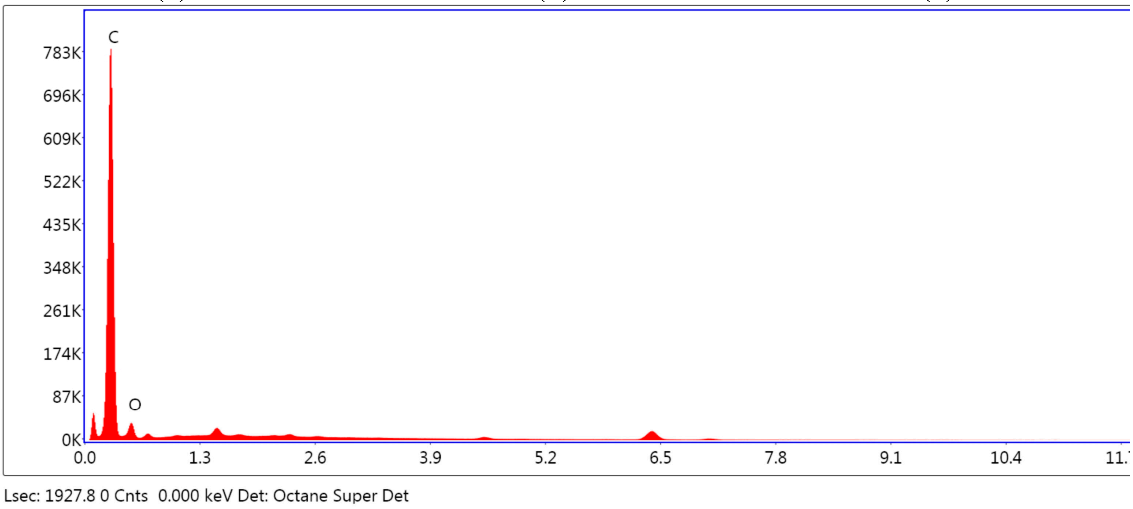

(d)

Fig. S3. The quantitative EDS element mapping of GO I (a); carbon C K (b); oxygen O K (c) and EDS spectrum (d).

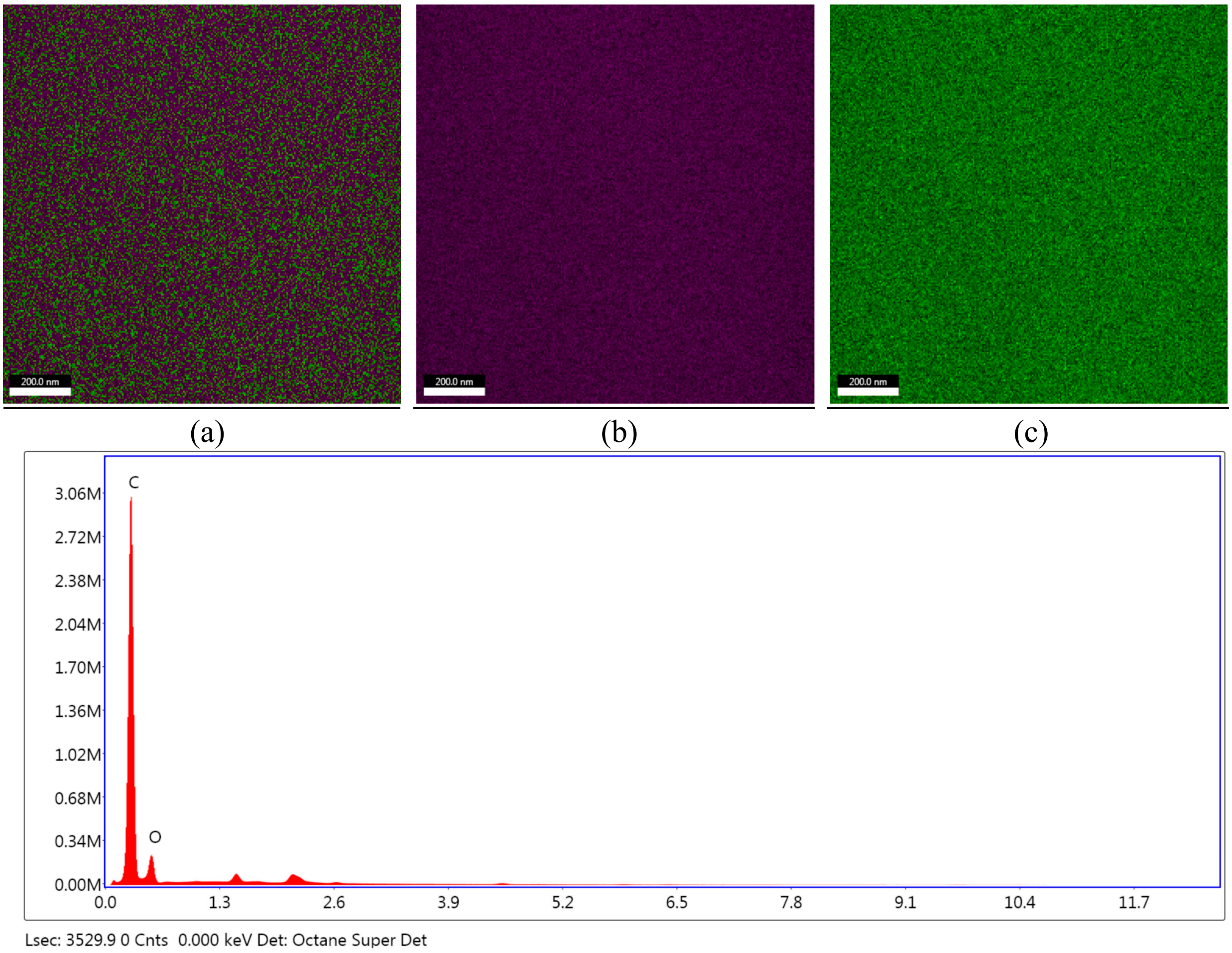

(d)

Fig. S4. The quantitative EDS element mapping of GO II (a); carbon C K (b); oxygen O K (c) and EDS spectrum (d).
